# Cell and Gene Therapies: European View on Challenges in Translation and How to Address Them

**DOI:** 10.3389/fmed.2018.00158

**Published:** 2018-05-28

**Authors:** Cécile F. Rousseau, Romaldas Mačiulaitis, Dariusz Śladowski, Gopalan Narayanan

**Affiliations:** ^1^Voisin Consulting Life Sciences, Paris, France; ^2^Committee for Advanced Therapies (CAT), European Medicines Agency (EMA), London, United Kingdom; ^3^Committee for Medicinal Products for Human Use (CHMP), European Medicines Agency (EMA), London, United Kingdom; ^4^Institute of Physiology and Pharmacology, Lithuanian University of Health Sciences, Kaunas, Lithuania; ^5^Department of Transplantology and CBT, Medical University of Warsaw, Warsaw, Poland; ^6^Voisin Consulting Life Sciences, Camberley, United Kingdom

**Keywords:** Advanced Therapy Medicinal Products, European Medicines Agency, translational challenges, scientific advice, regulatory agencies

## Abstract

Advanced therapy medicinal products (ATMPs), *i.e*., cell and gene therapy products, is a rapidly evolving field of therapeutic development. A significant proportion of the products are being developed by academia or small/medium-sized enterprises (SMEs). The many challenges in translation posed by this class of products include aspects covering: manufacturing, non-clinical development plan as relevant to clinical trial, marketing authorization, and reimbursement. In this context, the term translation refers to the relevance of non-clinical data in relation to how it impacts on appropriate and efficient clinical development. In order to successfully overcome these challenges, a clear understanding of the requirements and expectations of all the stakeholders is critical. This article aims to cover the potential challenges related to such translation and suggested approaches to find solutions based on experience and learnings from the perspective of European Union. While commercial challenges have a significant impact on the ATMPs in general, it is considered outside the scope of this article. However, by adopting a strong scientific basis for translation as suggested in this article, it is likely such an approach would help rather than harm successful real world clinical use of ATMPs.

## Background

Advanced therapy medicinal products (ATMPs), covering cell and gene therapy medicinal products and tissue-engineered products in the EU, is a rapidly growing area of medicines development. They are regulated in the EU as medicinal products but under a specific regulation applicable, *i.e*., (EC) No 1394/2007 as they are based on new and highly innovative technologies (*e.g*., genetically modified cells, various vectors from viral origin) (1).

A significant proportion of the products are being developed in academic settings or by small/medium-sized enterprises (SMEs) (2). Despite the high number of such products being under development, only few of them have been approved and even fewer have successfully reached patients. There have been 897 applications in Europe (EudraCT) referring to 519 unique clinical trials, referring to ATMP between 2009-2015 (3). There are many factors that contribute to the challenges in developing these products in order achieve successful clinical use. One such challenge is the successful translation of the non-clinical (NC) (also often referred as pre-clinical) evidence to achieve clinical relevance. While the main objectives of medicines regulation and the role of the relevant European experts is to ensure safe and effective products are approved, there is a general recognition that translational challenges posed by ATMPs requires a tailored approach when planning the life-cycle development of this class of products.

The Committee for Advanced Therapies (CAT) at European Medicines Agency (EMA) was established to ensure that the relevant expertise is available in regulatory decision making to support and evaluate these products. The role of national regulatory agencies as well as the EMA committees such as CAT is critical to ensure that the potential of these products are realized in practice. In order to support these novel products and optimize their development and assessment, several guidelines have been developed by the EMA in collaboration with relevant regulatory experts within the EU (4).

**Figure 1:**
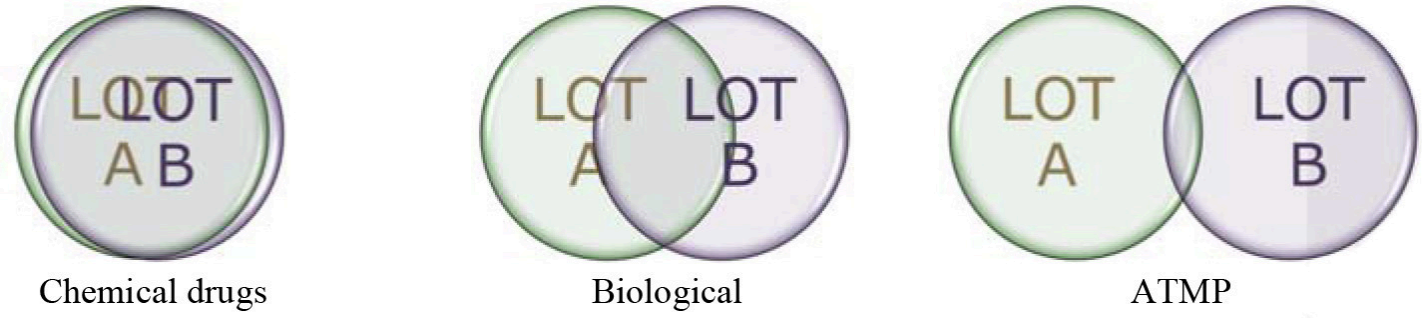
Lot-to-lot comparison for medicinal products.

There is general recognition that ATMPs are much more complex entities than small molecules and therapeutic proteins (Figure 1). Key to successful development requires a thorough understanding of the quality aspects, also commonly referred to as “Chemistry, Manufacturing, Controls” (CMC) in order to effectively translate such knowledge toward ensuring successful NC to clinical translation and successful clinical outcome. Subsequent development should be jointly planned and developed by NC and Clinical groups with full understanding of quality aspects and the potential impact on the target patient population (5).

If used with a device, aspects related to devices regulation should be planned for. If in doubt, early dialogue with regulatory agencies will help to a proper classification of the combination product, and will also enable appropriately tailored planning of NC development.

One of the biggest challenges of the ATMP development remains in the translational specificities from R&D to market authorization. The objectives of NC assessments are comparable between conventional medicinal products and ATMPs, but strategies differ (Table 1). The NC investigation for ATMPs is recommended to start with a risk based approach (RBA) in planning, which is a unique feature in the ATMP regulation (6). RBA aims to enable planning for relevant experiments for establishment of safety and efficacy, including first-in-human trial, market authorization and post-authorization follow-up. RBA also contributes to design more relevant NC studies in compliance with Reduce, Refine and Replace (3Rs) principle. Risks and risk factors are specific for the product and intended clinical use. They should be carefully determined by an integrated approach between CMC/NC/Clinical experts. The guideline on risk-based approach [EMA/CAT/CPWP/686637/2011, (6)] further expands on this aspect.

**Table 1:** Translational specificities for ATMPs.

**Translational aspects**	**Conventional medicinal product, including biological**	**ATMP**
Pharmacodynamics		
Proof-of-concept	Obligatory	Essential, if relevant animal
Mode-of-action	Obligatory	models are available
Off-target	Less defined	
Pharmacokinetics	Obligatory	Distribution
Dose to humans	Alloscaled	Alloscaling absent
SD acute toxicity	Important	
SD chronic toxicity	Not always relevant	Often relevant
MD chronic toxicity	Essential, GLP	
AESI	Very supportive	Often empirical

*AESI, adverse effects of special interest; MD, multiple dose; MoA, Mode of Action; PD, Pharmacodynamics; PK, Pharmacokinetics; PoC, Proof-of-Concept; SD, single dose*.

Translational challenges can further complicate development due to changes in product manufacturing between the R&D scale and clinical grade requiring evidence of comparability. One procedure that might enable early regulatory assessment is a Certification procedure (7). This could facilitate translational activities, enabling easier and faster preparation of the marketing authorization application (MAA).

## Challenges in translation

### Proof-of-concept/mode-of-action

The most frequent issue for cell and gene therapy products is to find the relevant animal model(s) to establish a NC proof-of-concept (PoC) and demonstrating a mode-of-action. In particular, the NC evidence should not mislead. A combination of *in vitro, ex vivo*, and *in vivo* studies could be beneficial in the absence of relevant animal models. Use of homologous model(s) if comparable to the intended clinical population could strengthen evidence despite the limitations in interpretations.

Absence of relevant models for evaluating the mode-of-action could hamper interpretations of both PoC and efficacy studies. Replacement of NC proof-of-concept and mode-of-action tests by clinical data could be acceptable for market authorization. Bridging from external similar developments might also be acceptable for market authorization, if justified.

### Dose translation

Dose translation is one of the biggest challenges for ATMPs. An example of such challenge is illustrated by the clinical study outcomes published by Kochenderfer et al. (8) on CAR-T cells. They showed that from the time of infusion to the time of peak blood levels, anti-CD19 CAR T cells acquired a more differentiated phenotype. The observed rapid decreases in patient blood CAR-positive T cells might be partially explained by the acquisition of more differentiated phenotypes. However, the relative importance of peak blood CAR-positive T-cell levels *vs*. sustained persistence of blood CAR-positive T cells remains unknown (8). The importance of the levels of blood CAR-positive T cells does not appear to be established and seems in general to be unknown (8). It is possible that a more important indicator of effectiveness in treating a cancer indication could be the number of CAR-positive T cells infiltrating the tumor mass. NC evaluation may show limitation in such assessment. Therefore, dose translation might be based on experiences from other relevant studies, combination of *in vitro* and *in vivo* studies. Data collected from literature for similar products can help to plan the NC and clinical doses while keeping in mind the impact of any differences between products. Learning from data reported for similar type of ATMP product might be used in combination with all CMC and NC data available to establish a dose range for Phase I clinical trial. This implies that quality characteristics are also comparable in such situations.

Experience with allometric scaling is limited, but equivalent surface area dosage conversion factors might be informative. Relevance of fixed dosage regimen for product with dividing and differentiating potential is generally unknown.

### NC biodistribution

GLP based NC evaluation for all safety related studies is normally required for any drug product. However, because of lack of relevant animal model(s) and/or the intended route of administration, strict application of GLP could be challenging. This is generally recognized by the regulatory agencies. On a case by case basis, regulators may consider use of non-GLP studies if the model used is justified as relevant and the study well-documented. Developers should in such case ensure the high quality of the protocol, the product (if possible same quality as the one intended for the clinical use, including sterilization and packaging if applicable) and data recorded and reports if the full GLP status cannot be maintained during the whole NC development plan. Potential of pharmacokinetic/pharmacodynamic assessment is often under-employed and thus developers miss opportunity to benefit from dose response analyses. Seeking early dialogue with the regulators to discuss the NC development plan would avoid the risk of using a non-relevant model. Alongside with ATMP product development, universal tracking systems for cell therapy products are also being developed. Such technologies could be considered for autologous cell-based medicinal products (CBMP) for example.

### Translational toxicity studies

Pivotal toxicity studies should normally be as per GLP. Any deviation is a risk that will need to be assessed and agreed to by regulatory agency experts *via* special EMA procedures such as Scientific Advice (9) or as part of ATMP Certification procedure (7). The impact on biodistribution and safety due to differences in immunity between animals and human (induced and/or pre-existing *e.g*., AAV vector products) needs to be evaluated and interpreted appropriately to ensure the translation is meaningful and beneficial for planning clinical development.

### Mutual challenges and possible next steps

The translational experience in ATMP area is constantly evolving (Table 2). Ideally, positive and negative experiences from all stakeholders should be made publicly available. While published documents such European Public Assessment Report (EPAR) can help, there are currently insufficient number such documents. Developers should think more creatively about how to develop either good new generic animal models (such as transgenic animal models or animal homolog models) for both efficacy and toxicity studies, or product-tailored animal models (*e.g*., disease models, immunocompromised models, or homolog models) as they might be more relevant to the product to be characterized. The development of the new generic animal models is a challenge for any developer. One possible way to address this issue would be a proactive combined private public partnership approach, such as EU Horizon 2020 type of activities (*i.e*., COSME, EU programme for the Competitiveness of Enterprises and SMEs, 2.3 bln EUR). One key element is that the combination of several models might actually be the best approach to address the safety concerns that may arise from the ATMP nature. Learning from the first successful ATMP products being used on patients might contribute to such knowledge database.

**Table 2:** Mutual challenges and possible next steps for ATMPs.

**Translational aspects**	**Challenge**	**Possible next steps**
PoC/MoA	Absence of relevant animal model	Call for generic studies, *e.g*., for H2030?
PK/PD	Limited to predominantly distribution studies	Comprehensive approaches should be developed *e.g*., via BMQ?
Dose translation	Alloscaling absent	Systematic generic analyses needed?
Chronic SD/MD toxicity	Exemplar RBA cases are limited	Periodic updates? Encourage public development of relevant toxicity animal models?
GLP environment	Not always followed	New procedure at NCA/EMA SA level?

*BMQ, Biomarker qualification; H2030, Horizon 2030; MD, multiple dose; MoA, Mode of Action, PD, Pharmacodynamics; PK, Pharmacokinetics; PoC, Proof-of-Concept; RBA, Risk Based Approach; SD, single dose*.

## From bench to clinic

It is acknowledged that the complexity of ATMPs will likely increase the time and cost of the NC development plan. However, by collecting more supportive NC evidences, it is envisaged that it could make clinical development more efficient and contribute to successful clinical use of the product.

The basic science aspect of product development can take 3–5 years (average duration of a scientific grant). A complete development prior to marketing authorization could take 10 or more years. A historical example of development which bears similarities to an ATMP is the bone marrow transplantation. This first successful use of stem cells from unrelated donor transplant took place in 1957, winning Nobel Prize in physiology or medicine in 1990, after more than 30 Years. This is even more complex in case of combined ATMPs, with a medical device in addition. All components need to be characterized separately and in the combination, which increases dramatically complexity, costs, and time of development.

How to ensure successful development? Due to the intrinsic complexity of these products, clinical development should be an integrated approach encompassing CMC/NC/Clinical teams. This approach would ensure that potential benefits and risks for the intended use is clearly understood, estimated and planned for during the entire clinical development. NC development plan has significant limitations as described before *e.g*., conventional ADME tests are usually not applicable. As already mentioned, defining the dose-response relationship *in vitro*/*in vivo* for the intended effect and for the potential safety concerns could be very difficult, and developers will have to innovate to address such concerns. Biodistribution, migration, persistence, engraftment, proliferation, differentiation, insertion, transitory effect, duration of exposure are key questions for which classical evaluation methods do not apply to ATMP.

The proof-of-concept for any medicinal product is the pillar of any further NC and clinical development. To demonstrate the product potential, relevant model(s) of the disease *in vitro, ex vivo*, and/or *in vivo* can be used, keeping in mind that maybe a combination of several of these models might lead to the information regarding the ATMP characterization. With regards to validity of models, it is even more challenging for ATMP as they might need uncommon model(s) to be used such as immunocompromised, immunosuppressed, knockout, transgenic, humanized, and/or disease animal model. Whatever the animal model chosen, developers will need to keep in mind the limitations of the models and the relevant information that the model could provide.

Immunocompromised or immunosuppressed models for example will allow xenogeneic studies but will obviously limit the immunological analyses; moreover, depending on their origin (*e.g*., specific animal strain, or chemically induced), these models might exhibit some metabolic reactions that should be taken in account while analyzing the data. In oncology studies, humanized mouse models have the advantage to recreate in a “semi-controlled” environment with the potential to reflect the encounter between the tumor, the human immune cells, and the ATMPs product, but one should keep in mind that the “uncontrolled” part remains a murine environment and the applicable limitations. The residual murine innate immune system continues to impede human cell engraftment. To address this, various knockout strains are therefore being created to further reduce murine innate immunity (10). For cell therapy product involving autologous cells, developers could consider investigating animal homolog models if relevant.

Identification of biological activity markers, such surface marker panel comparable for in both human and animal cells, and relevant to clinical intended use such as route of administration and frequency could help. This will allow demonstrating the comparability of the animal product to the human product and leveraging the NC data collected from the homologous animal model. If those products appear to be comparable, the animal version will support certain assessment such as biodistribution, as long as the tracking method does not modify the product. An interesting example is the usefulness of *in vitro* tests in case of living skin dressings (substitutes) used in case of treatment of skin burns and no healing wounds. The use of *in vitro* skin models similar to the products have been intensively studied as a model of human skin which has been accepted as scientifically validated model of human skin *in vivo*. It has been subjected to extended scientific peer review.

The following example illustrates the challenges in the evolution of an ATMP. In March 2000, European Commission unanimously endorsed the statement of validity of *in vitro* skin model for *in vitro* irritation testing. In 2008 at the European Commission in Brussels, the non-Commission members of the ECVAM Scientific Advisory Committee (ESAC) Joint Research Centre, European Commission endorsed *in vitro* human skin models of human skin (EpiDerm and SkinEthic RHE) as a valid alternative to *in vivo* animal tests for irritancy testing (11). The model has been subsequently accepted by OECD (12). The accumulated knowledge and experience helps promote the acceptance of a developed skin substitutes for wound treatment (13). Establishment of a European Registry of patients treated with hospital exempted products could further help successful development of novel therapies.

In summary, NC development plan for ATMPs should take a risk based approach, prepared in coordination with CMC and clinical groups. *In vitro, ex vivo*, and/or *in vivo* model(s) will require complete validation to ensure translation to clinical, keeping in mind the clinical intended use and route of administration as well as the importance of positive and negative controls.

## How EU regulatory agencies help development of ATMPs

There has been a constant rise in the number of ATMPs being developed as indicated by a significant increase in the applications for classification and scientific advice requests, 293 and 288, respectively (14).

Among the many interactions ATMP developers can have with the EMA, two procedures in particular could help with translation. These are:

1. Certification, which is unique as it is only available for ATMPs and carried out by EMA/CAT, and
2. Scientific Advice, through CHMP/SAWP in consultation with EMA/CAT.

Both these procedures provide opportunities to evaluate NC data as well as proposal for strategy for translation. For the latter, it is also possible to seek advice from national agencies in addition.

Certification procedure for CMC and/or NC data is an incentive from EMA for SMEs. This allows assessment of early CMC and NC data. Such procedure has a fast turnaround time (90 days), and certificate may attract investments.

Scientific Advice can be given on any scientific question, CMC, NC, and clinical. EMA and/or National Competent Authorities' feedback could be sought through scientific advice at any time point of the development. Post-marketing advice is also available. Developers will through this process get broad advice on development and also suitability for *e.g*., conditional approval and approval under exceptional circumstances. Such communications remain confidential.

**Figure 2:**
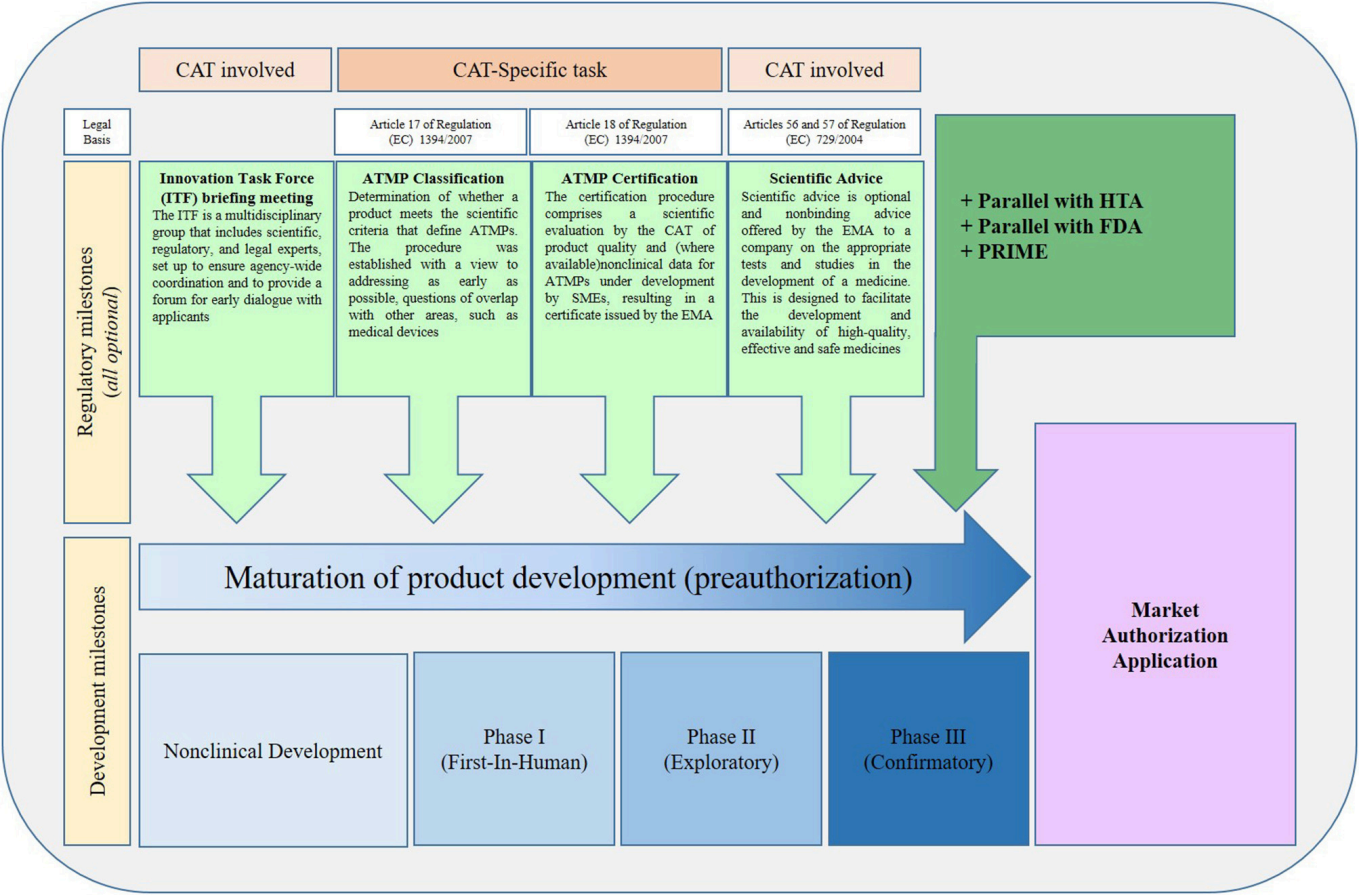
Regulatory pathways for ATMPs in Europe. The usual sequence in which procedures are requested by applicants. Note that all procedures can be requested at any time during development. ATMP, Advanced Therapy Medicinal Product; CAT, Committee for Advanced Therapies; EMA, European Medicines Agency; FDA, Food and Drug Administration; HTA, Health Technology Assessment body; PRIME, Priority medicine procedures; SME, Small and Medium-sized Enterprise. [Adapted from Maciulaitis et al. (2), (15)].

ATMPS developers are encouraged to have early communication with regulatory bodies (national competent authorities, EMA, notified bodies), and by using the following communication tools [Figure 2, (2)].

EMA helps developing ATMPs by providing several guidelines (4). However, not all products can be covered by specific guidelines and because of limited experience so far, guidelines tend to cover mainly general principles. EMA encourages scientists, investors, physicians, pharmaceutical companies, technical support, and regulators to create working groups to help create new and specific guidelines.

## Conclusion

While regulations are established and guidelines are available to facilitate successful translation, it is important to note that the developers need to fully understand the product in relation to the intended use and plan prospectively the approach for successful translation. While regulators can provide valuable input and advice, this can at best complement the knowledge and planning of the developers who should have in-depth understanding and knowledge of the product. As such, developers should be able to justify their study designs, test model(s) (*in vitro, ex vivo*, and/or *in vivo*), and/or absence of specific studies *etc*.

Using an integrated approach covering CMC/NC/Clinical aspects is key for any pharmaceutical product but it is even more critical for ATMPs. (16) A science- and risk-based approach to ATMP development, such as Quality by Design (QbD), remains challenging for ATMPs. However, adaption of QbD principles such as the development of an adapted Control Strategy based on Risk Assessment and identification of the critical steps and critical raw materials is possible and encouraged (17). Because these model(s) will most likely be innovative and the size of the ATMP lots produced most of the time are limited, developers are strongly advised to initiate dialog with the agency(ies) as early as possible through scientific advice procedure.

EMA/CAT and National Competent Authorities are part of the same European System: their activities are complementary and provide the developers with relevant feedback aiming to improve the development plans toward a successful outcome. Early dialogue with EMA and National agencies as appropriate is strongly encouraged for successful such development.

## Author contributions

All the authors (CR, RM, DŚ, and GN) equally contributed to the article writing.

## Conflict of interest statement

CR PhD is employed by Voisin Consulting Life Sciences, Paris, France. GN is employed by Voisin Consulting Life Sciences, Camberley, United Kingdom. The other authors declare that the research was conducted in the absence of any commercial or financial relationships that could be construed as a potential conflict of interest.
